# Supplementary material for: Instant Cascara Beverage as a Neuroimmune Modulator of the Brain–Gut Axis: Sex-Dependent Effects in Healthy Rats
Source: Int J Mol Sci. 2025 Nov 3;26(21):10691. doi: 10.3390/ijms262110691 (PMC12609599; doi:10.3390/ijms262110691)
Supplement: Supplementary file 1 [file ijms-26-10691-s001.zip › ijms-3767786-supplementary.pdf]

International Journal of *Molecular Sciences*

MDPI

Supplementary Material

Instant Cascara Beverage as a Neuroimmune Modulator of the Brain-Gut Axis: Sex-Dependent Effects in Healthy Rats

Paula Gallego-Barceló[1,2], Yolanda López-Tofiño[1,2,3,4], Laura López-Gómez[1,2], Gema Vera[1,2,5], Ana Bagues[1,2,5,6], Jesús Esteban-Hernández[7,8], María Dolores del Castillo[2,9], José Antonio Uranga[1,2], Raquel Abalo[1,2,4,5,10] *

[1] Department of Basic Health Sciences, Faculty of Health Sciences, University Rey Juan Carlos (URJC), 28922 Alcorcón, Spain; paula.gallego@urjc.es (P.G.-B.); yolanda.lopez@urjc.es (Y.L.-T.); laura.lopez.gomez@urjc.es (L.L.-G.); gema.vera@urjc.es (G.V.); ana.bagues@urjc.es (A.B.); jose.uranga@urjc.es (J.A.U.)

[2] High Performance Research Group in Physiopathology and Pharmacology of the Digestive System (NeuGut-URJC), University Rey Juan Carlos (URJC), 28922 Alcorcón, Spain; mdolores.delcastillo@csic.es

[3] Working Group of Basic Sciences on Neuropathic Pain of the Spanish Pain Society, 28046 Madrid, Spain

[4] Working Group of Basic Sciences on Cannabinoids of the Spanish Pain Society, 28046 Madrid, Spain

[5] Associated I+D+i Unit to the Institute of Medicinal Chemistry (IQM), Scientific Research Superior Council (CSIC), 28006 Madrid, Spain

[6] High Performance Research Group in Experimental Pharmacology (PHARMAKOM-URJC), University Rey Juan Carlos (URJC), 28922 Alcorcón, Spain

[7] Department of Medical Specialties and Public Health, Area of Preventive Medicine and Public Health, Faculty of Health Sciences, University Rey Juan Carlos (URJC), 28922 Alcorcón, Spain; jesus.esteban@urjc.es (J.E-H)

[8] High-Performance Research Group on Environmental Risks for Health and the Environment, University Rey Juan Carlos (URJC), 28922 Alcorcón, Spain

[9] Food Bioscience Group, Department of Bioactivity and Food Analysis, Instituto de Investigación en Ciencias de la Alimentación (CIAL) (CSIC-UAM), Calle Nicolás Cabrera 9, 28049 Madrid, Spain

[10] Working Group of Basic Sciences on Pain and Analgesia of the Spanish Pain Society, 28046 Madrid, Spain

* Correspondence: raquel.abalo@urjc.es; Tel.: +34-91-488-88-54

Academic Editor: Firstname Lastname

Received: date
Revised: date
Accepted: date
Published: date

Citation: To be added by editorial staff during production.

Copyright: © 2025 by the authors. Submitted for possible open access publication under the terms and conditions of the Creative Commons Attribution (CC BY) license (https://creativecommons.org/licenses/by/4.0/).

Body weight, solid and liquid intake

During the three-week study, male control rats gained 35% of their baseline weight, while females showed no significant changes (Figure S1A). Male control rats consumed 23 g/rat/day of food, significantly more than females (16 g/rat/day; $p < 0.0001$, Figure S1B). For liquid intake (Figure S1C), males consumed 33 ml/rat/day, and females consumed 29 ml/rat/day, with no significant differences. Exposure to the IC beverage did not result in any statistically significant changes in weight, food intake, or liquid intake compared to controls, except for a significant difference in liquid intake between males and females exposed to IC (thus, dependent on sex, and not on the type of beverage consumed).

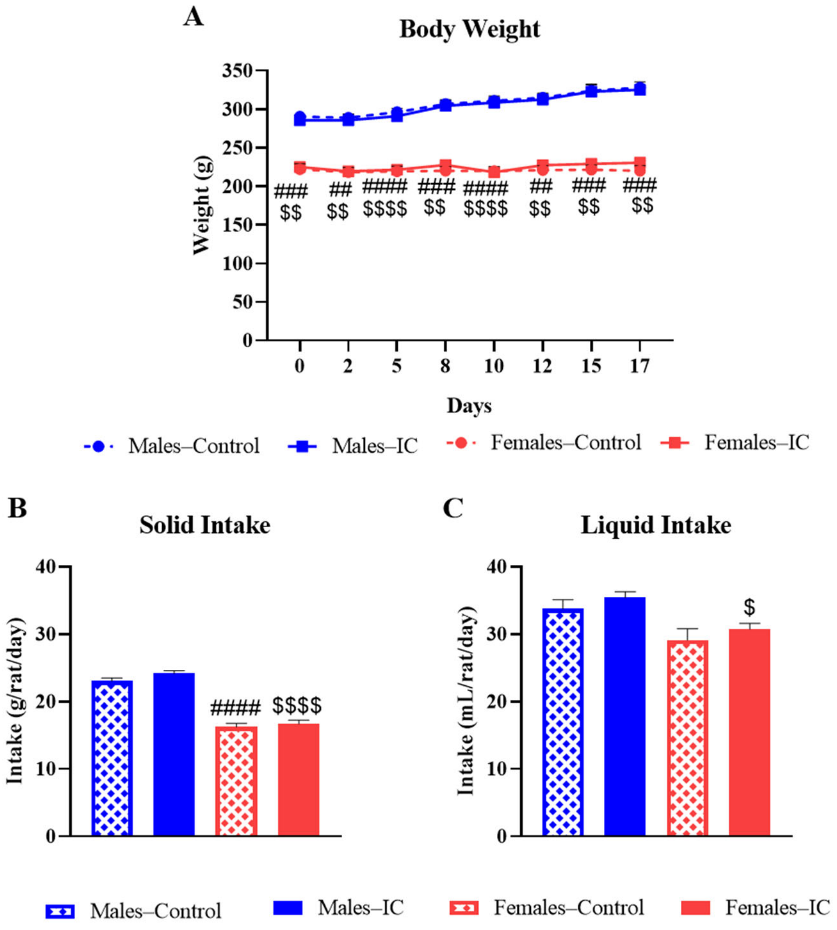

Figure S1. Effect of regular exposure to Instant Cascara (IC) beverage on body weight gain and on the solid and liquid intake of rats of both sexes. Body weight gain (A) and the overall mean solid (B) and liquid (C) intakes by each experimental group are shown. These parameters were recorded in the four experimental groups, distributed according to sex and the administered beverage (IC or water): Males–Control, Males–IC, Females–Control and Females–IC. Data represent mean ± SEM (standard error of the mean). N = 9 animals per group. Sex-dependent statistically significant changes: ## $p<0.01$, ### $p<0.001$, #### $p<0.0001$ [Females–Control vs Males–Control]; $ $p<0.05$, $$ $p<0.01$; $$$$ $p<0.0001$ [Females–IC vs Males–IC]. One-way ANOVA followed by Tukey's post-hoc test or Kruskal–Wallis' test followed by Dunn's post-hoc multiple comparison test as appropriate.

Vaginal cytology smear in female rats

The distribution of estrous cycle phases in females (proestrus/estrus vs. metestrus/diestrus) across two cohorts under different experimental conditions is shown in Table S2. Females were predominantly in proestrus/estrus phase when they were used for the behavioral studies, and in the control group of cohort 2 (used for colon sample analyses after sacrifice), whereas distribution was more balanced in the IC group of this cohort. Nevertheless, no statistically significant differences were observed between control and IC groups at any of the occasions when this was evaluated.

Table S1. Distribution of female animals according to the phase of the estrous cycle during the studies.

			**proestrus/estrus (%)**	**metestrus/diestrus (%)**
**Cohort 1**	Somatic Sensitivity and Locomotor Activity	Control	66%	34%
		IC	78%	22%
	Visceral Sensitivity	Control	89%	11%
		IC	89%	11%
**Cohort 2**	Sacrifice	Control	82%	18%
		IC	45%	55%

Two experimental groups, distributed according to the administered beverage (IC or water), were used: Females–Control and Females–IC. In cohort 1 (n= 9 per /group), vaginal cytology samples were taken from females after the somatic sensitivity experiments (Von Frey Test, PAM) and locomotor activity experiments conducted in the second week of the study. In the third week, cytology was performed after the visceral sensitivity study. In cohort 2 (n= 6-9 per /group), cytology was conducted prior to sacrifice of the females. Data represent mean ± SEM. Fisher's exact test.

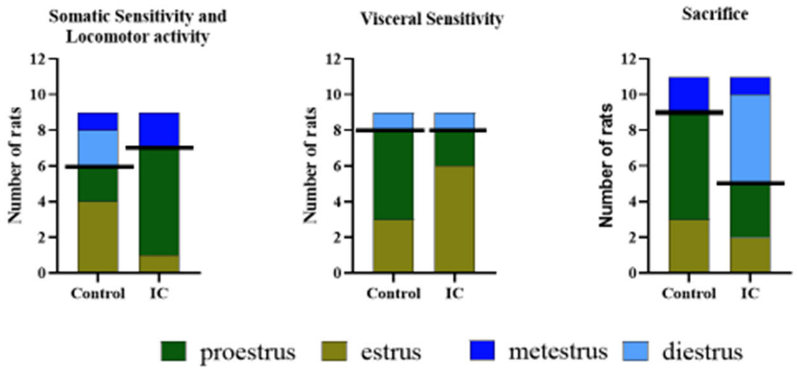

Figure S2. Estrous cycle distribution in control and IC females. In cohort 1 (n = 9 per group), vaginal cytology was performed after somatic sensitivity (Von Frey, PAM) and locomotor activity experiments (week 2), and after visceral sensitivity testing (week 3). In cohort 2 (n = 6–9 per group), cytology was performed prior to sacrifice. Bars show the number of females in each estrous phase (proestrus, estrus, metestrus, diestrus). For analytical purposes, the phases were grouped into two categories: proestrus/estrus and metestrus/diestrus, with the black horizontal line indicating the division between these two categories. Data are expressed as mean ± SEM. Fisher's exact test.

Nutritional Composition

Table S2. Nutritional Composition of Instant Cascara Beverage (10 mg/ml).

Components		Instant Cascara Beverage (10 mg/ml)
**Carbohydrates (mg/ml)**		4.748
**Total fiber (mg/ml)**		1.832
**Lipids (mg/ml)**		0.058
**Protein (mg/ml)**		0.625
**Free amino acids (mg/ml)**	Aspartic acid	0.019
	Glutamic acid	0.021
	GABA	0.003
**Magnesium (mg/ml)**		2.084×10^{-3}
**Sodium (mg/ml)**		26.658×10^{-3}
**Potassium (mg/ml)**		228.4×10^{-3}
**Calcium (mg/ml)**		5.478×10^{-3}
**Caffeine (mg/ml)**		0.139
**Phenolic content (mg eq. CGA/ml)**		0.89
**Chlorogenic acids (mg/ml)**		1.07-1.26
**Melanoidins (mg/ml)**		1.5

This table presents the nutritional composition of the Instant Cascara beverage at a concentration of 10 mg/ml. The data include macronutrients, minerals, bioactive compounds, and other relevant components, such as amino acids of interest (aspartic acid, glutamic acid, and GABA), expressed in mg/ml or equivalent units, as specified. Note: The phenolic content is expressed in mg equivalents of chlorogenic acid per ml (mg eq. CGA/ml), representing the concentration of phenolic compounds quantified based on their equivalence to chlorogenic acid. GABA: gamma-aminobutyric acid. Table adapted from Gallego-Barceló et al., 2024 [24].
